# Mentioned in Dispatches

**Published:** 1915-06-26

**Authors:** 


### MENTIONED IN DISPATCHES.

In a dispatch received by the Secretary of State for War from the Field-Marshal Commanding-in-Chief, British Forces in the Field, the "names of those" recommended "for gallant and distinguished service in the field" include the following members of the Medical and Red Cross Services. There are 3,772 names in the entire list:—

### Army Service Corps (Attd. R.A.M.C.).

Bean, T. 22608 Sgt. J.; Coombes, 18853 Sgt. F. W.; Maley, 3595 Sgt. P. F.; Saunders, T. 16278 Actg. Sgt. F. L.; Tysall, T. 11930 Sgt. A.; Irwin, 769 Lce.-Cpl. J.; Littler, 032744 Lce.-Cpl. A.; Williams, T. 24838 Lce.-Cpl. J.; Jefferson, T/2/016753 Driver B.; Sutcliffe, T. 18839 Pte. G.

### Medical Service and R.A.M.C.

Bowlby, Tempy. Col. Sir A. A., K.C.M.G., F.R.C.S.
Burghard, Tempy. Col. F. F., M.D., F.R.C.S.
Dawson, Tempy. Col. Sir B. E., K.C.V.O., M.D.
Gray, Tempy. Col. H. M. W., M.B., F.R.C.S. (Edin.).
Herringham, Tempy. Col. Sir W. P., M.D.
Lister, Tempy. Col. W. T., M.B., F.R.C.S.
Meek, Col. J., M.D.
Makins, Tempy. Col. Sir G. H., K.C.M.G.,C.B.,F.R.C.S.
Moynihan, Tempy. Col. Sir B. G. A., M.B., F.R.C.S.
Wright, Tempy. Col. Sir A. E., M.D., F.R.C.S.I., F.R.S.
Young, Col. C. A.

Ainsworth, Maj. R. B.; Archibald, Qrmr. and Hon. Capt. W. N.; Barnard, Qrmr. and Hon. Lt. A. P.; Bartlett, Maj. R. S.; Bell, Capt. W. J. E., M.B.; Birch, Qrmr. and Hon. Lt. E.; Bond, Capt. A. H.; Bostock, Maj. J. S., M.B.; Boyce, Capt. W. W.; Boyle, Lt.-Col. M., M.B.; Brown, Maj. R. T., M.D.; Browne, Capt. C. G.; Buckley, Qrmr. and Hon. Capt. E. J.; Burney, Capt. W. H. S.; Burnham, Maj. H. H., attd. 2nd Canadian Artillery Brig.

Cagney, Tempy. Lt. P., M.B.; Cairns, Lt. J. W., M.D. (T.F.); Cannon, Lt. J. W., M.B. (S.R.); Carr, Qrmr. and Hon. Lt. J. (T.F.); Carter, Capt. H. St. M., M.D.; Chesney, Lt. W. McM., M.B. (S.R.); Clarke, Lt. T. W., M.B. (S.R.); Conway, Qrmr. and Hon. Lt. T. D.; Coplans, Capt. M., M.D. (T.F.); Cowey, Maj. R. V.; Craig, Lt. D. D., M.B.; Crawford, Lt.-Col. G. S., M.D.; Crean, Capt. T. J., V.C. (Res. of Off.); Cree, Tempy. Lt. R. E., M.B.

Dalrymple, Tempy. Capt. J.; Darling, Capt. W., M.B., F.R.C.S. (Edin.); Dawson, Capt. G. F., M.B.; Devonald, Lt. A. E. L. (T.F.); Du Cros, Tempy. Hon. Capt. G. H.; Dugdale, Qrmr. and Hon. Lt. H. (T.F.); Duka, Tempy. Capt. A. T., D.S.O.

Escott, Qrmr. and Hon. Lt. E. W. J.; Fielding, Maj. T. E., M.B.; Foster, Capt. J. R.; Franklin, Maj. R. J.; Gale, Capt. R., M.B.; Galloway, Tempy. Lt. R. W., M.B.; Gilchrist, Lt. A. J. (S.R.); Glynn, Tempy. Lt. A. S., M.B.; Goddard, Maj. C. E., M.D. (T.F.); Green, Qrmr. and Hon. Maj. J.; Green, Qrmr. and Hon. Lt. R. H.; Grenfell, Qrmr. and Hon. Lt. T.; Harding, Maj. D. L., F.R.C.S.I.; Harris, Qrmr. and Hon. Lt. G. W. (T.F.); Hart, Tempy. Lt. C. H., M.B.; Haycraft, Tempy. Lt. J. B., M.B.; Herrick, Lt.-Col. H.; Hewson, Capt. F. M., F.R.C.S.I.; Hill, Tempy. Lt. F. T.; Hincks, Lt. A. C. (T.F.); Houston, Capt. J. W., M.B.; Howell, Capt. F. D. G.; Howell, Capt. H. L.; Ingram, Tempy. Lt. T. L.; Irvine, Capt. A. E. S.; James, Tempy. Lt. P. W., M.D.; Janion, Tempy. Lt. H. G.; Jones, Capt. J. B., M.B.; Just, Tempy. Lt. T. H., M.B.

Kavanagh, Capt. E. J., M.B.; Kelly, Lt.-Col. J. F. M., M.B.

Lane, Capt. J. W., M.D.; Langstaff, Lt.-Col. J. W.; Le Bas, Tempy. Lt. D.; L'Estrange, Maj. E. F. Q.; Lightstone, Lt. H. (T.F.); Lindsay, Lt. A. B., M.B.; Lister, Tempy. Lt. W. H.; Lloyd-Jones, Capt. P. A., M.B.; Lovell, Lt. A. G. H., M.B., F.R.C.S.; Lunney, Qrmr. and Hon. Capt. A.

McCarthy, Capt. W. H. L., M.D. (Special Reserve); McCurry, Lt. W. T. (S.R.) (killed); Macglashan, Capt. K. B., M.D., F.R.C.S. (Edin.) (S.R.); Mackie, Lt. D., M.B. (S.R.); McLaughlin, Capt. J. N., M.D. (S.R.); Maclean, Tempy. Lt. I. C., M.D.; McLean, Lt. W. F., M.B. (S.R.); McSheehy, Capt. O. W., M.B.; Martin, Maj. J. F., M.B.; Martyn, Capt. S., M.B. (T.F.); Miller, Lt. W. A., M.B. (S.R.); Moore, Tempy. Lt. E. H., D.S.O., M.B.; Morris, Lt. E. M. (T.F.); Morrison, Qrmr. and Hon. Capt. A. (Res. of Off.).

O'Brien, Capt. C. W.; Offord, Qrmr. and Hon. Maj. E. P.

Packard, Qrmr. and Hon. Lt. J. T.; Page, Capt. C. M., M.B., F.R.C.S. (S.R.); Pallant, Maj. S. L.; Pemberton, Lt. H. S., M.B. (S.R.); Poe, Lt.-Col. J., M.B.; Preston, Lt. R. A., M.B.; Priestly, Lt. J. G., M.B.

Richards, Maj. F. G. (killed); Richards, Tempy. Capt. O., M.D.; Robinson, Capt. T. T. H., M.B.; Rogers, Maj. H., M.B.; Rogers-Tillstone, Lt.-Col. J. M. (T.F.).

Sargent, Tempy. Hon. Lt.-Col. P., M.B., F.R.C.S.; Seelley, Tempy. Lt. E., M.B.; Shaw, Capt. J. J. McI., M.B.; Short, Qrmr. and Hon. Maj. J. B.; Slayter, Lt.-Col. E. W., M.B.; Smith, Tempy. Lt. A. C. S.; Smith, Tempy. Lt. P.; Somervell, Lt. L. C.; Spackman, Qrmr. and Hon. Maj. H.; Stirling, Capt. A. D., M.B.; Stubbs, Capt. J. W. C., M.B.; Symons, Lt.-Col. F. A., D.S.O., M.B.

Thorne, Tempy. Lt. F. J., M.B.; Thurston, Lt.-Col. H. S.; Trist, Capt. J. R. R. (S.R.); Turnbull, Tempy. Lt. D. C., M.B. (died of wounds); Turner, Capt. C. H.

Vellacott, Capt. H. F., F.R.C.S. (S.R.).

Ward, Qrmr. and Hon. Lt. H. A.; Wear, Lt.-Col. A. E. L., M.D.; Webb, Lt.-Col. A. L. A.; Wedd, Lt. B. H., M.D.; Wells, Capt. A. G.; West, Maj. J. W., M.B.; Whitehead, Capt. N. T., M.B.; Wilkinson, Tempy. Lt. R. F.; Wilson, Tempy. Lt. C. McM., M.D.; Winter, Capt. H. G.; Woolfenden, Tempy. Lt. H. F., M.D., F.R.C.S.; Wood, Capt. J. L.; Woodhouse, Tempy. Lt. P. R., M.B.; Worthington, Maj. Sir E. S., Knt., M.V.O.; Worthington, Capt. F., M.B.; Wylie, Lt. T. W., M.B. (S.R.).

Andrews, 14050 Qrmr.-Sgt. W.; Argent, 7438 Actg. Sgt.-Maj. W.; Arnold, 7527 Pte. P. F.; Avery, 2217 Actg. Cpl. F.; Bardswell, 19106 Actg. Cpl. G. F.; Barron, 19812 Sgt. F. P.; Batchelor, 10179 Pte. P.; Benson, 7174 Pte. J. F.; Blewitt, 9115 Pte. W. T.; Bowler, 17964 Sgt. W.; Boxall, 19665 Sgt. H. G.; Burdett, 7742 Actg. Cpl. G.; Bush, 15027 Staff-Sgt. W.; Butler, 18566 Cpl. H.; Cartwright, 7685 Pte. J.; Chase, 7247 Pte. S. J.; Clark 5190 Actg. Cpl. A.; Cook, 5018 L.-Cpl. W. C.; Dady, 18222 Staff-Sgt. A.; Davis, 20589 Pte. W. J.; Deakin, 19818 Pte. J. G.; Dewberry, 16115 Sgt.-Maj. E. B.; Emmerson, 7695 Pte. F.; Evans, 5145 Pte. A.; Floyd, 20732 Pte. H.; Forbes, 14924 Staff-Sgt. J. G. A.; Gibbs, 8269 Sgt.-Maj. G. A.; Gillespie, 10254 Sgt.-Maj. A.; Godfrey, 14452 Cpl. F.; Greenwood, 20796 Pte. H.; Gregory, 284 Sgt. A. O.; Gregson, 16205 Qrmr.-Sgt. T.; Hamer, 2690 Pte. W. H.; Harris, 18443 Sgt. G.; Harrison, 9751 Pte. J.; Hayne, 8633 Pte. E. W.; Herbert, 18964 Sgt. G. W.; Horrigan, 1675 Pte. P.; Hutchings,

(*Continued on page* vi.)

